# Optimizing Patient Access to Orphan Medicinal Products: Lessons from Central and Eastern Europe

**DOI:** 10.3390/jmahp13020024

**Published:** 2025-05-26

**Authors:** Tomasz Kluszczynski, Bertalan Nemeth, Magdalena Władysiuk, Marcin Czech, Maria Kamusheva, Nicolae Fotin, Sandra Rose, Tomáš Doležal, Rok Hren

**Affiliations:** 1 ACESO Healthcare Consulting, 03-719 Warsaw, Poland; 2 Business School, Warsaw University of Technology, 02-008 Warsaw, Poland; 3 Syreon Research Institute, 1142 Budapest, Hungary; 4 Department of Epidemiology and Preventive Medicine, Jagiellonian University, 31-007 Cracow, Poland; 5 Department of Pharmacoeconomics, Institute of Mother and Child, 01-211 Warsaw, Poland; 6 Faculty of Pharmacy, Medical University of Sofia, 1431 Sofia, Bulgaria; 7 Media Kompass, 023808 Bucharest, Romania; 8 Chiesi Pharmaceuticals GmbH, 1010 Vienna, Austria; 9 iHETA, 120 00 Prague 2, Czech Republic; 10 Value Outcomes, 120 00 Prague 2, Czech Republic; 11 Institute of Mathematics, Physics, and Mechanics, 1000 Ljubljana, Slovenia; 12 Faculty of Mathematics and Physics, University of Ljubljana, 1000 Ljubljana, Slovenia

**Keywords:** orphan medicinal products, Central and Eastern Europe, health technology assessment, rare diseases, patient access, regional collaboration, healthcare disparities

## Abstract

This study examines patient access to orphan medicinal products (OMPs) in Central and Eastern Europe (CEE) over the past five years, focusing on seven countries: Bulgaria, Czechia, Hungary, Poland, Romania, Slovakia, and Slovenia. While these jurisdictions have undergone rapid healthcare transformations, significant disparities in OMP access persist compared to Western Europe. This study aimed to address this gap by identifying barriers and enablers to optimize patient access to OMPs in a sustainable and equitable manner. A mixed-methodology approach was utilized, combining systematic literature reviews, in-depth interviews, and advisory board insights. Perspectives were gathered from a wide range of stakeholders, including policymakers, payers, academia, industry associations, and patient advocacy groups. Additionally, the study incorporated data from CEE-specific initiatives to triangulate findings and evaluate barriers, enablers, and best practices in OMP access. The analysis identified sub-optimal OMP access across most CEE countries, marked by prolonged delays and lower reimbursement rates compared to Western Europe, with Slovenia and Czechia as notable exceptions. Key barriers include limited awareness, inadequate health technology assessment (HTA) frameworks, insufficient financing mechanisms, underutilization of novel access schemes, and fragmented patient engagement. Conversely, enablers include the presence of rare disease policies, OMP-specific HTA frameworks, and patient-inclusive decision-making processes.

## 1. Introduction

Rare diseases are life-threatening or chronically disabling conditions that reduce the lifespan and worsen the quality of life of patients and impose a substantial burden on carers [1,2]. Timely diagnosis and the selection of an appropriate therapeutic strategy can not only improve care for these patients but can also reduce or offset the direct and indirect costs associated with supportive treatment or a lack of treatment [3]. Pricing and reimbursement processes for orphan medicinal products (OMPs) play a crucial role in patients’ access to optimal treatment, whilst innovation-oriented policies can provide an additional incentive for the industry to continue or increase their research and development (R&D) efforts in areas where such treatments are still unavailable [4,5].

It is essential that the costs for OMPs are reimbursed through public funds, considering that their price is markedly higher compared to treatments for more common diseases [6,7]. To achieve this, a transparent and evidence-based approach to pricing and reimbursement processes is necessary, with the provision of scientifically supported evidence on the cost-effectiveness, the economic burden of therapy, and, of equal importance, the consideration of other factors such as the availability of alternative treatments, the severity and disabling nature of the disease, and the moral and ethical aspects [6,7]. Notably, over one-third of OMPs target oncology indications, including rare cancer subtypes [8]. However, their high revenues and rapid growth raise concerns about the long-term sustainability of funding [5].

Central and Eastern Europe (CEE) faces unique hurdles in ensuring equitable access to OMPs. The region’s healthcare systems are marked by economic constraints, variable infrastructure, and fragmented policies [9,10,11,12,13], all of which impact the availability and affordability of OMPs. Moreover, the disparities in healthcare spending across CEE countries—ranging from 6.5% to 10% of GDP [14]—highlight the financial challenges that influence policy decisions. Despite these limitations, recent years have seen progress, with several CEE countries adopting innovative policies and frameworks to enhance access to OMPs. By 2024, nearly all CEE countries had introduced some form of rare diseases plan, either integrated into broader drug policy documents, such as Bulgaria’s National Health Strategy or Slovakia’s Medicines Policy, or as stand-alone initiatives, as seen in Czechia, Poland [15,16,17], and Romania. While these plans offer a valuable framework for policymakers and healthcare providers, there is currently conflicting evidence linking their adoption to measurable improvements in patient access to OMPs, suggesting that other factors may play a more critical role. The variable impact of policy frameworks highlights the need to better understand common success factors.

The objectives of this study are as follows:

- Assess the evolution of healthcare systems in CEE, i.e., examine the development of healthcare systems in CEE over the past five years, with a particular focus on patient access to OMPs;
- Identify barriers and enablers, i.e., analyze both shared and country-specific challenges affecting OMP access, identifying key obstacles as well as factors that facilitate improvements;
- Develop actionable recommendations, i.e., provide evidence-based strategies to optimize OMP availability across the region, addressing systemic inefficiencies and proposing targeted interventions.

## 2. Materials and Methods

**Figure 1:**
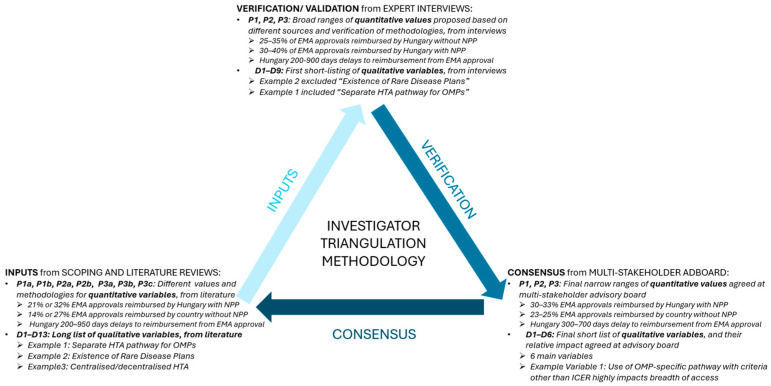
Investigator triangulation methodology applied in this study. The triangulation methodology integrates inputs from scoping and literature reviews, expert interviews, and multi-stakeholder advisory board consensus. The process refines quantitative values and qualitative variables to enhance the robustness and reliability of findings related to OMP access in CEE.

In this study, we focused on seven CEE countries—Bulgaria, Czechia, Hungary, Poland, Romania, Slovakia, and Slovenia—which collectively account for 19% of the EU population and contribute over 1.8 trillion USD in GDP. For each country, a dedicated health technology assessment (HTA) expert was engaged, with eligibility requiring active involvement in HTA as of 7 December 2023 and demonstrated expertise in the field. To comprehensively evaluate the five-year evolution of patient access to OMPs in CEE, this study employed a rigorous, multi-phased, mixed-methods approach. Ethical approval was obtained for all interviews, ensuring informed consent and confidentiality. The research adhered to principles of transparency and stakeholder inclusivity, aligning with standards in social science research [18]. As shown in Figure 1, a robust triangulation was applied [19], accounting for limitations such as data variability across countries, potential stakeholder biases, and conflicting inputs from the literature reviews; these challenges were further mitigated through diverse stakeholder engagement and cross-validation of findings. The methodology employed in this study was not just a framework for data collection and refining numerical values but a crucial mechanism for gaining insight into the complexity of OMP access. By leveraging investigator triangulation—combining literature reviews, expert validation, and multi-stakeholder consensus—this study navigated the inconsistencies inherent in cross-country healthcare evaluations. Our methodology was thus designed in somewhat unorthodox fashion by combining six distinct phases to enable a detailed evaluation of the barriers and enablers influencing OMP access in the CEE region.

### 2.1. Phase 1: Literature Review (September–October 2023)

A systematic literature review was conducted using PubMed, Scopus, and regional databases to identify peer-reviewed studies, reports, and policy papers published in the last five years. The search employed Medical Subject Headings (MeSH) terms and keywords, such as “orphan medicinal products”, “orphan drugs”, “Central and Eastern Europe”, “health technology assessment”, and “rare diseases”. Grey literature, including governmental and NGO reports, was also included. Data extraction focused on barriers, enablers, and outcomes related to OMP access.

### 2.2. Phase 2: Multi-Round Stakeholder Interviews (October–December 2023)

Semi-structured interviews were conducted in three iterative rounds to capture stakeholder perspectives. In Round 1, policymakers and HTA professionals were interviewed to understand systemic and regulatory landscapes. In Round 2, clinicians, payers, health economists, and industry representatives provided insights into operational challenges and innovative solutions. In Round 3, patient advocacy groups and academic researchers contributed perspectives on patient-centric approaches and data gaps. The interviews followed an open-ended format, allowing for in-depth exploration of topics and iterative refinement of findings. Transcripts were thematically coded using NVivo software version 15 and emergent themes were triangulated with findings from the literature and scoping reviews. Details of the participants and their affiliations are provided in Table 1.

### 2.3. Phase 3: Advisory Board of HTA Experts (7 December 2023)

An advisory board of nine regional HTA experts, i.e., authors of this paper (T.K., B.N., M.W., M.C., M.K., N.F., S.R., T.D., and R.H.), validated the preliminary findings and formulated actionable recommendations. The three sessions focused on (i) reviewing synthesized findings from Phases 1 and 2, (ii) identifying best practices and innovative approaches, and (iii) drafting recommendations to improve equity and sustainability in OMP access.

### 2.4. Phase 4: Scoping Review (November 2023–February 2024)

A complementary scoping review explored broader themes and identified knowledge gaps. This phase incorporated insights from non-indexed sources, including conference proceedings, white papers, and regional initiatives such as the G.A.P. (Gearing Up Access Proposal) tool [20] and EFPIA W.A.I.T. (Waiting to Access Innovative Therapies) [21] Indicator publications. The review mapped regulatory frameworks, reimbursement policies, and HTA methodologies across the seven CEE countries.

### 2.5. Phase 5: Data Integration and Analysis (March–October 2024)

Quantitative data on OMP availability, reimbursement timelines, and HTA processes were systematically integrated with qualitative insights to provide a comprehensive analysis. EMA-authorized OMP lists were extracted, while EFPIA W.A.I.T. Indicators and national reimbursement records were used to validate findings. The primary outcomes analyzed included OMP coverage (as a percentage of EMA approvals, both with and without Named Patient Programs, NPPs) and reimbursement delays (measured as days from EMA authorization). Results were presented as ranges and validated through consultations with advisory board HTA experts. Six key dimensions influencing OMP access were evaluated (Table 2). Particular attention was given to analyzing case studies that could potentially highlight successful strategies, such as the implementation of tailored HTA pathways and the integration of patient perspectives in decision-making processes.

### 2.6. Phase 6: Advisory Board’s Final Consensus on Barriers, Enablers, and Key Recommendations (October–December 2024)

The advisory board of HTA experts reviewed the final draft and provided feedback through virtual interviews or written comments. A rapid literature review identified potential new developments in OMP policies, such as the publication of new rare disease plans or appraisal methodologies, ensuring the study remained current. Based on the data and analyses, barriers and enablers were identified and key recommendations were proposed by the advisory board. Only those barriers, enablers, and recommendations achieving full consensus among board members were included.

## 3. Results

### 3.1. Overview of Patient Access to OMPs Across CEE

**Figure 2:**
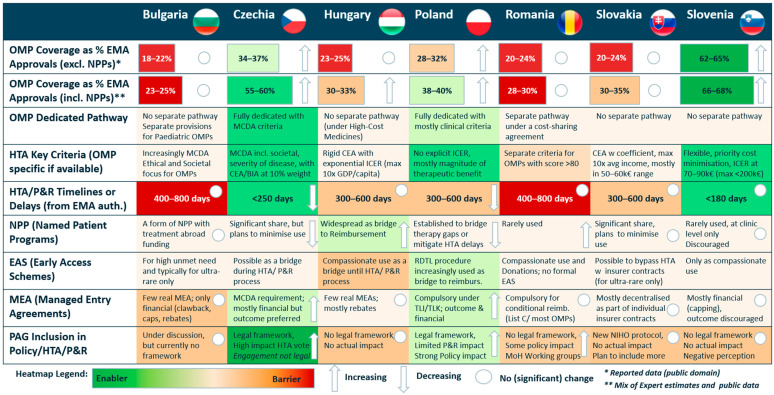
Comparison of patient access to orphan medicinal products (OMPs) in Central and Eastern Europe (CEE), highlighting three key outcomes: OMP coverage as a percentage of EMA approvals (with and without Named Patient Programs) (first and second row) and reimbursement delays (in days) (fifth row). The heatmap also evaluates six dimensions influencing access, including health technology assessment (HTA) pathways (third row), tailored criteria for OMPs (fourth row), the use of Named Patient Programs (NPPs) (sixth row), Early Access Schemes (EASs) (seventh row), and managed entry agreements (MEAs) (eighth row), and the inclusion of patient advocacy groups (PAGs) in decision-making processes (ninth row). The information presented is a synthesis of multiple data sources, systematically integrated through the triangulation methodology, which was instrumental in refining quantitative values and qualitative variables related to OMP access in CEE. The three outcomes and six dimensions were derived from a structured process involving literature reviews (Section 2.1 and Section 2.4), multi-stakeholder discussions (Section 2.2), and expert interviews (Section 2.5), ensuring alignment with real-world healthcare system dynamics.

Figure 2 provides an overview of patient access to OMPs across CEE, focusing on three critical outcomes: OMP coverage as a percentage of EMA approvals (with and without NPPs) and time delays in reimbursement from EMA authorization [22]. OMP coverage varies notably across the region. Excluding NPPs, Slovenia achieves the highest access rates at 62–65%, comparable to Western European leaders like Germany (84%), Denmark (71%), Italy (70%), Austria (69%), and Sweden (63%) (source: validated IQVIA weight indicator data; status: 2022) and surpassing all other Western countries. Including NPPs has the largest impact in Czechia, increasing OMP rates from 34–37% to 55–60%, whereas in Bulgaria, Romania, Slovakia, and Hungary, OMP rates even with NPPs remain below 35%. Reimbursement timelines reveal further differences, with Slovenia achieving the shortest delays (<180 days), while Bulgaria and Romania experience substantial delays ranging from 400 to 800 days.

One of the primary contributors to delays in OMP access is the requirement for international reference pricing, as well as, in the cases of Romania and Bulgaria, the additional HTA referencing timelines and budget constraints for agreements with the payer. For most CEE countries, international reference pricing stipulates that the HTA and reimbursement process cannot begin or be finalized until several reference countries have completed their own pricing assessments. This dependency introduces inherent delays, compounded in Romania and Bulgaria by their unique HTA referencing procedures [23,24]. Specifically, these countries mandate that the technology must first be evaluated by other Western European HTA bodies—three for Romania and at least one of four (United Kingdom, Germany, Sweden, and France) for Bulgaria—before the local pricing and reimbursement process can even commence. Further delays arise during the negotiation phase for MEAs with payers, which can extend the process by up to two years.

Slovenia stands out as an exception within the CEE region, employing a pragmatic approach to international reference pricing that significantly reduces delays. Slovenia determines the maximum allowed price by referencing the minimum price in three countries. However, unlike other nations, if pricing data from all three countries are not available in a timely manner, Slovenia allows the reference to be based on just two or even a single country. This flexibility accelerates the HTA process, price setting, and MEA negotiations. In some instances, this approach has enabled Slovenian patients to gain access to OMPs earlier than patients in traditionally fast-adoption countries such as Germany, Denmark, and Austria (typically, between 180 and 220 days) (source: validated IQVIA weight indicator data; status: 2022).

The six dimensions in Figure 2 reveal the impact of policy and operational factors on OMP access in CEE. Dedicated HTA pathways ensuring comprehensive evaluations for OMPs could improve accessibility, as observed in Czechia and Poland, while other countries lack such frameworks, leading to delays. Notably, Slovenia lacks a dedicated pathway yet achieves exceptional OMP coverage through its abovementioned pragmatic approach to HTA processes. Tailored HTA criteria, such as Czechia’s multi-criteria decision analysis (MCDA) [25], incorporate broader societal and ethical considerations, enhancing decision-making. Conversely, rigid cost-effectiveness analyses (CEAs), prevalent in some countries, appear to restrict access to OMPs.

NPPs bridge access gaps during HTA evaluations, as seen in Czechia and to some extent in Hungary and Slovakia, though it remains uncertain if NPPs alone can adequately meet patient demand. While they offer an alternative access pathway, NPPs often introduce increased uncertainty and impose additional administrative burdens on both patients and clinicians, including extensive paperwork and procedural complexities which are more demanding compared to standard reimbursement processes.

Early Access Schemes (EASs) have proven effectiveness in providing interim access to therapies [26] but are only moderately employed in the CEE, with some implementations in Poland. Meanwhile, outcome-based MEAs, which have the potential to mitigate high cost barriers effectively, remain underutilized across most CEE countries [27]. Financial agreements tend to dominate, with a limited adoption of innovative outcome-based models.

The inclusion of patient advocacy Groups (PAGs) enhances patient-centric approaches, with Czechia and Poland leading in formal PAG engagement during HTA and policy decision-making processes. However, many other CEE countries exhibit minimal involvement of PAGs [28,29]. Interestingly, Slovenia achieves exceptional OMP coverage despite the absence of formal patient inclusion, reflecting unique jurisdictional efficiencies. For further insights, Appendix A presents case studies from Czechia and Poland, illustrating the implementation of tailored HTA pathways. Appendix B features a case study from Czechia that focuses on the integration of patient inclusion in decision-making processes.

### 3.2. Advisory Board’s Consensus on Barriers, Enablers, and Key Recommendations for Facilitating Patient Access to OMPs Across CEE

On 18 December 2024, the advisory board members identified and reached consensus on five key barriers and three critical enablers affecting patient access to OMPs in CEE, as outlined in Table 3. Building on these analyses, a total of seven recommendations were developed and unanimously agreed upon, as detailed in Table 4.

## 4. Discussion

The findings of our study emphasize the necessity of adopting rare disease frameworks and tailored HTA pathways [30] to improve patient access to OMPs across the CEE region. Our comprehensive methodology provided a detailed understanding of OMP access barriers and enablers in the CEE region, as compared to the rest of Europe [31], forming a foundation for both evidence-based and actionable recommendations. By addressing systemic barriers and proposing targeted strategies, this research aims to support policymakers, healthcare stakeholders, and patient advocacy groups in their efforts to enhance access to OMPs. Through its comprehensive approach, this study seeks to contribute to improved healthcare equity and outcomes for rare disease patients in CEE.

### 4.1. Healthcare System Evolution

Over the past five years, CEE healthcare systems have undergone transformations aimed at improving access to high-cost and innovative therapies, including OMPs. The region’s progress is often characterized as “leapfrogging”, where incremental advancements have been facilitated by a paradigm shift, with healthcare stakeholders adopting the perspective of “healthcare as an investment” rather than a cost.

### 4.2. Strengthening HTA Frameworks

Czechia demonstrates how strategic investments in HTA pathways and rare disease frameworks can yield improvements in OMP coverage; the integration of MCDA into HTA processes provides a useful model for balancing clinical efficacy, cost-effectiveness, and societal impact. To address inadequate HTA frameworks, CEE countries should adopt tailored methodologies that account for the unique characteristics of OMPs (e.g., [32]), including societal and patient-centric perspectives.

### 4.3. Patient-Centric Policies

Involving patients in decision-making processes is an important step toward equitable healthcare. Systematic patient inclusion, as demonstrated in Czechia, ensures that policies reflect the lived experiences and priorities of those directly affected by rare diseases. Expanding educational programs and advocacy initiatives could empower patient organizations to contribute meaningfully to HTA and reimbursement discussions. There is a high urgency to raise awareness of the actual burden of rare diseases [33]. To integrate patients into HTA process successfully, other CEE countries ought to consider following a proven step-by-step process, such as the one Czechia underwent between 2017–2022. This process can be best described as the 5Ds (5 consecutive steps): Diagnose, Define, Design, Develop, and Deploy. Firstly, the Czech MoH, aided by the industry association (AIFP), diagnosed the status quo and mapped all relevant patient organizations in the area of rare diseases. Secondly, optimal governance and funding mechanisms were defined to ensure efficient processes and prevent any conflict of interest. Next, all patient organizations that complied with the rules of governance and funding were aggregated into an umbrella organization and a comprehensive educational program was designed to create patient experts, capable of engaging in clinical, HTA, and socio-economic topics. Finally, the new HTA OMP pathway, with patient experts having voting rights, was effectively deployed in 2022, which has led to demonstrable improvements to OPM patient access.

### 4.4. Sustainable Financing Mechanisms

The establishment of dedicated funds, such as Poland’s Medical Fund, represents the first step toward addressing financial constraints. However, broader adoption of outcome-based MEAs and innovative financing models is needed to ensure long-term sustainability.

### 4.5. Study Limitations

This study has several limitations. It excluded countries from the Western Balkans and Eastern Europe, such as Ukraine and Belarus, which might have provided additional insights into barriers and enablers at different stages of healthcare development. While the involvement of nine HTA experts on the advisory board may have influenced the findings, the broader stakeholder engagement in Phase 2 likely kept a balanced perspective. Overall, the advisory board served as a pragmatic approach to establishing a foundation for developing recommendations to enhance patient access to OMPs.

An important additional limitation of this study is that the evidence was gathered from various sources that were not always fully compatible in terms of data integration. Despite employing appropriate mitigation methodologies, there remains the possibility of unaccounted evidence gaps, which may impact the final conclusions.

### 4.6. Enhancing Regional Collaboration

Collaboration among CEE countries offers an opportunity to pool resources, share best practices, and establish regional data networks. By developing joint registries and leveraging shared platforms for HTA assessments, CEE countries could reduce redundancies, enhance data quality, and improve decision-making processes. Regional initiatives could also facilitate joint negotiations with manufacturers, ensuring better pricing and access agreements.

### 4.7. Future Research

Future research could benefit from additional quantitative analysis on OMP approval rates, pricing disparities, and patient outcomes. Due to data limitations, we were unable to assess approval rates in terms of unsuccessful reimbursement filings, nor did we have access to OMP pricing across individual jurisdictions. Additionally, data comparing patient outcomes between countries with and without reimbursed OMPs were not available. Addressing these important variables in future studies could offer a more comprehensive understanding of the financial and clinical implications of OMP access, ultimately strengthening policy recommendations and healthcare strategies in the region.

This study identifies key barriers, such as limited HTA frameworks and financial constraints, alongside important enablers, including rare disease policies and patient advocacy efforts. While these factors are examined individually, their interactions warrant further exploration. The interplay between barriers and enablers is complex, as financial constraints may limit the effectiveness of rare disease policies, while strong patient advocacy can sometimes drive policy improvements despite HTA limitations.

## 5. Conclusions

In our study, we (i) assessed the evolution of healthcare systems in CEE, (ii) identified barriers and enablers affecting OMP access, and (iii) developed actionable recommendations. The evolution of healthcare systems in CEE over the past few years has demonstrated both progress and persistent challenges in ensuring patient access to OMPs. Despite systemic constraints, countries like Slovenia and Czechia have achieved prominent advancements through innovative policies. However, many barriers—such as fragmented data infrastructure, inadequate HTA frameworks, and limited financing models—remain prevalent across the region.

## Figures and Tables

**Table 1:** Detailed participant information for Phase 2: multi-round stakeholder interviews.

Country	Round 1: Policymakers and HTA Professionals	Round 2: Clinicians, Payers, Health Economists, and Industry Representatives	Round 3: Patient Advocacy Groups and Academic Researchers
DineBulgaria	Former Deputy Health Minister	Head of local ISPOR chapter, member of industry association ARPharM	Former Minister of Health and current patient ombudsman
Czechia	Current Deputy Health Minister	Senior employee of public insurance company VZP, head of industry association AIFP	Head of patient council (MoH advisory body)
Hungary	Former deputy head of OGYEI (HTA body)	Senior health economics expert, Syreon Institute	Managing partner of local HTA consulting company, former senior advisor to rare diseases patient organization
Poland	Former Deputy Health Minister	CEO of local HTA consulting company, head of local ISPOR chapter	Head of “umbrella” rare diseases patient organization, national consultant in neurology/lead for Polish rare diseases plan
Romania	Former head of National Agency for Medicines and Medical Devices (HTA body)	Former head of ARPIM industry association	Board member of EURORDIS and Head of “umbrella” rare diseases patient organization
Slovakia	Former deputy head of NIH (HTA body)	Head of local ISPOR chapter	Scientific advisor to rare diseases patient organization
Slovenia	Former senior expert from JAZMP (pricing body)	Senior health economics expert, Syreon Institute	Head of “umbrella” rare diseases patient organization

**Table 2:** Six key dimensions influencing orphan medicine product access.

	Dimension
1	Dedicated OMP HTA pathways: The existence and implementation of pathways tailored to OMPs.
2	Tailoring of HTA criteria to OMPs: Adaptations in assessment frameworks specific to the unique challenges of OMPs.
3	Use of Named Patient Programs (NPPs): Interim mechanisms for providing access during HTA evaluations.
4	Use of Early Access Schemes (EASs): Systems enabling OMP availability prior to formal reimbursement decisions.
5	Managed entry agreements (MEAs): Financial and outcome-based models designed to mitigate high-cost barriers.
6	Patient inclusion in HTA and reimbursement processes: The integration of patient perspectives in decision-making frameworks.

**Table 3:** Barriers and enablers in facilitating patient access to orphan medicinal products (OMPs) in Central and Eastern Europe (CEE). ICER—incremental cost-effectiveness ratio; MEA—managed entry agreement; HTA—health technology assessment; MCDA—multi-criteria decision analysis.

	Barrier
1	Low awareness: Low awareness of rare diseases and OMPs’ specificity, in terms of disease burden, unmet needs, and net costs, leading to limited societal solidarity and low priority in drug policy and financing.
2	Inadequate HTA: Methodologies are unadjusted to the unique nature of OMPs, requiring unattainable or partially unattainable evidence, often unable to appropriately assess the societal perspective, and use overly restrictive decision criteria, such as ICER thresholds.
3	Insufficient and inefficient financing models: Models are not delineated for OMPs and are often grouped with funds for other technologies and services, increasing competition for resources.
4	Sub-optimal use of novel access schemes: Schemes such as outcome-based MEAs or systematic Early Access Schemes (EASs) for potential breakthrough innovations are under-utilized, instead applying ‘cost-myopic’ financial and cost-containment measures.
5	Fragmented patient engagement: There is infrequent or unsystematized patient participation in HTA and reimbursement processes, with often inadmissible patient data inputs.
	Enabler
1	Rare disease policy frameworks: Setting out comprehensive and long-term guidance for patients, clinicians, payers, and policymakers, assigning clear priorities, proposing systemic solutions to existing barriers, and establishing multi-stakeholder collaboration platforms (plans for rare diseases and rare disease programs).
2	OMP-specific HTA: Using broad societal perspective, beyond cost effectiveness and budget impact, to appraise the true value of OMPs, either with MCDA or a MCDA “decision tree” process.
3	Patient empowerment and education: Developing legal frameworks, practical solutions, and a stepwise approach towards meaningful engagement and inclusion of patients and patient organizations in key HTA and reimbursement processes.

**Table 4:** Key recommendations for facilitating patient access to orphan medicinal products (OMPs) in Central and Eastern Europe (CEE). HTA—health technology assessment; PAG—patient advocacy group; PRO—patient-reported outcomes; R&D—research and development.

	Key Recommendation
1	Raising awareness: High urgency to raise awareness of the actual burden of rare diseases and to systematize possible patient access solutions based on the existing and novel best practices from CEE and broader EU.
2	Integrating rare disease care: Mapping and optimizing patient pathways by involving all medical specialists to improve care, diagnostic accuracy, treatment effectiveness, and overall patient support.
3	Regional collaborations: Exchange best practices, co-create solutions, create data networks and regional Centers of Excellence (CoEs), and implement policy frameworks for joint HTA and novel payment mechanisms.
4	Including and amplifying voice of patients: Intensify efforts to map, educate and empower PAGs to be optimally engaged in HTA and decision-making, utilizing the value of patient data (PRO, preferences, and patient experience).
5	Adopting best practices: Identify and map best practices, primarily from across the CEE and from wider Europe, to inform policy changes, financing models, and other immediately transferrable solutions (see key recommendation 3 above).
6	Promoting health equity: Through a series of papers/publications showcasing equity as an integral part of value-based healthcare and future-proofing CEE healthcare systems.
7	Incentivizing OMP R&D: Both at the EU level and specifically at the CEE level, by participating actively in the implementation of the EU Pharmaceutical Package, by addressing local barriers and by continuously creating local/regional initiatives.

**Figure A1:**
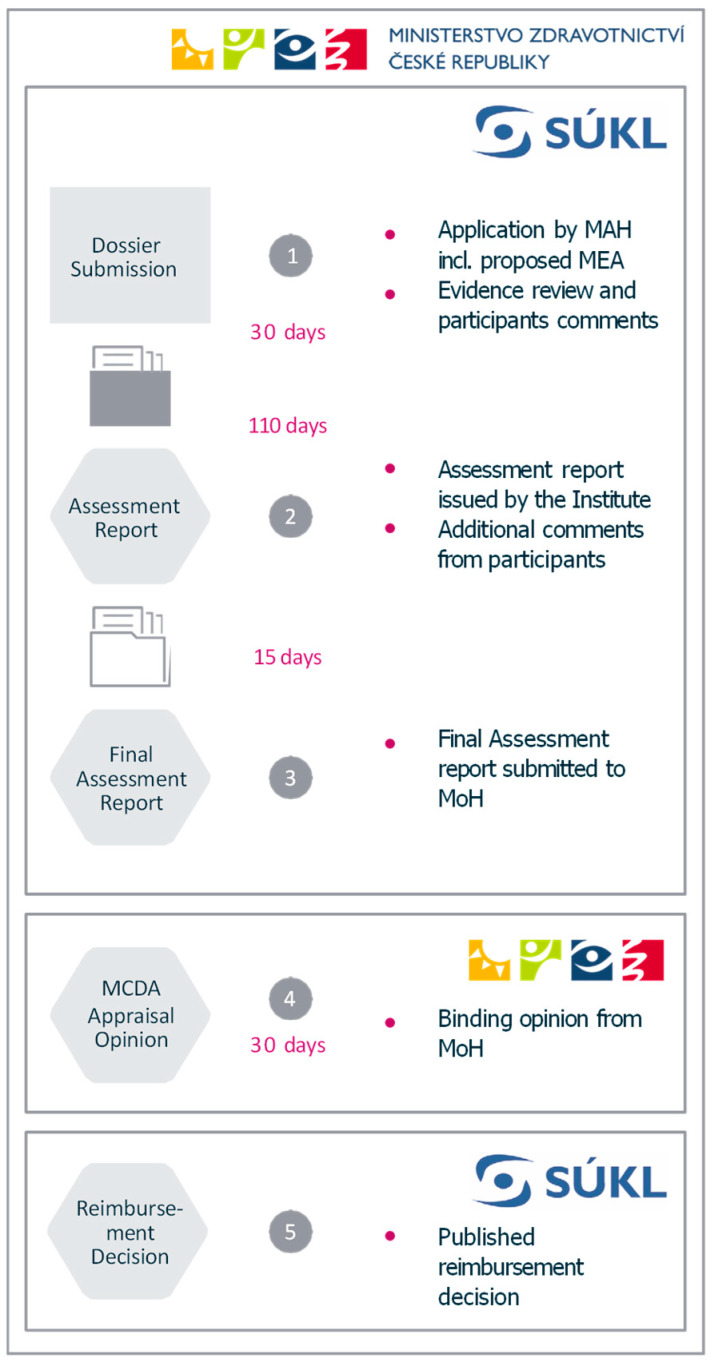
Implementation of OMP-specific HTA pathway in Czechia.

**Figure A2:**
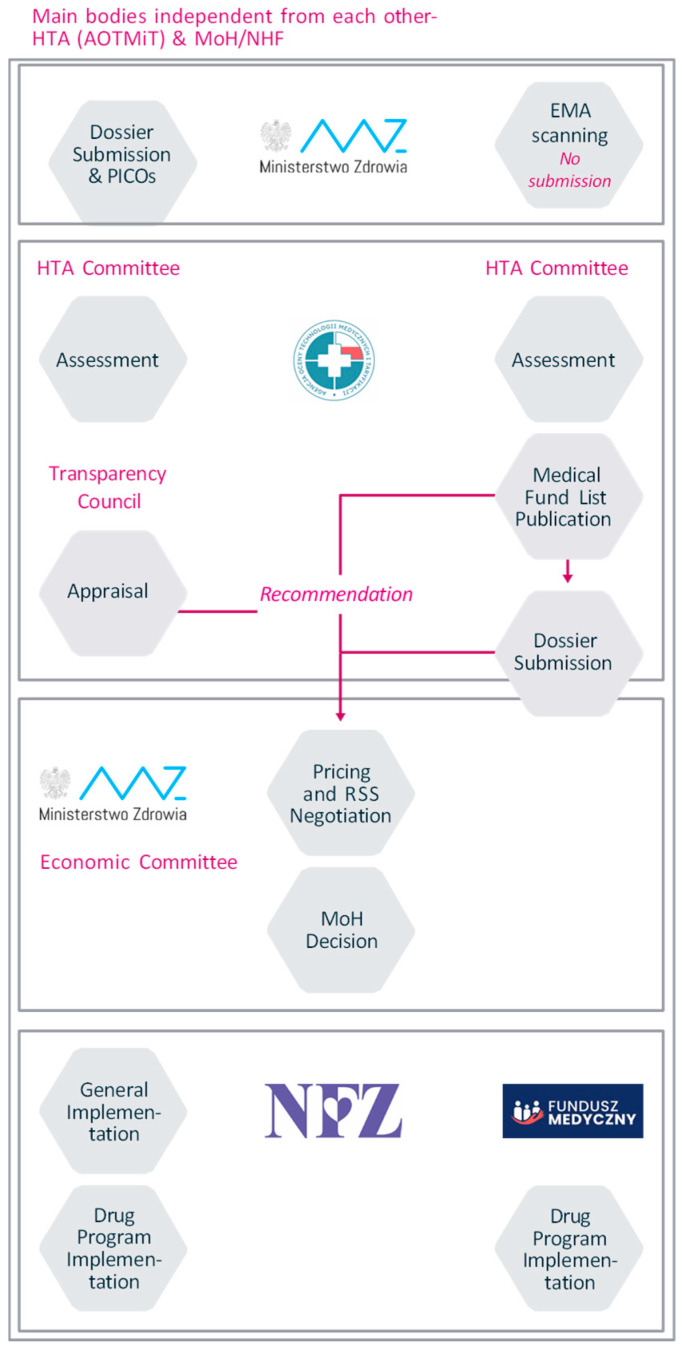
Implementation of OMP-specific HTA pathway in Poland.

## Data Availability

The data presented in this study are available upon request from the corresponding author.

## Abbreviations

The following abbreviations are used in this manuscript:

CEE	Central and Eastern Europe
HTA	health technology assessment
ICER	incremental cost-effectiveness ratio
MCDA	multi-criteria decision analysis
MEA	managed entry agreement
OMP	orphan medicinal product
PAG	patient advocacy group
PRO	patient-reported outcomes
R&D	research and development

## Appendix A. Case Studies from Czechia and Poland Illustrating the Implementation of OMP-Specific Pathways

Tailoring HTA pathways to the unique nature of OMPs is one of the success factors in achieving more equitable patient access. Czechia and Poland are the only two CEE countries where such a dedicated HTA pathway exists, though each country has adopted a different solution in terms of procedure, methodology, and decision criteria.

The Czech OMP-specific pathway is one of the first full MCDA methodologies implemented systematically in HTA assessment and appraisal, leading to a binding reimbursement recommendation. As shown in Figure A1, the Czech OMP process starts with a submission of application to the State Institute for Drug Control (SUKL) with relevant or required evidence. Relevant professional associations, PAGs, and health insurance funds are entitled to present evidence and make comments during the first 30 days. The SUKL performs a full assessment and initial appraisal of the evidence and publishes an Assessment Report summarizing the available information.

All participants have the right to comment on the Assessment Report within 15 days from its publication, after which the SUKL publishes a final Assessment Report and forwards it to the Ministry of Health and its Advisory Body. The Advisory Body consists of four stakeholders with equal voting rights (25% each): patient representatives, clinical experts, public health insurance funds, and a MoH/state representative. The advisory body critically evaluates the documents and (within 30 days) issues a binding opinion based on the decision-making criteria.

The Polish model, known as TLI/TLK, has been often likened to a type of ‘late horizon-scanning exercise’, occurring during or, more commonly, immediately after an EMA authorization procedure. In other words, the Polish HTA (AOTMiT) scans for promising technologies which have submitted an EMA application, performs their preliminary assessment prior to local dossier submission, and uses the results to invite/not invite the authorization holder to negotiate pricing, reimbursement, and MEAs.

In general, there are two alternative pathways for RD reimbursement in Poland: (i) The standard ICER-driven model linked to the general NHS drug budget and (ii) the TLI/TLK pathway with no explicit pharmacoeconomic requirement, linked to the special Medicines Fund. Since HTA data are not considered in the TLI/TLK pathway, the Transparency Council step, used for appraisals, is skipped or streamlined. Instead, National Consultants opine on the magnitude of the therapeutic value or unmet needs (Figure A2).

OMPs recommended in TLI/TLK for reimbursement are published in the Medicines Fund list, requesting the marketing authorization holder to submit a dossier within 1 year with a full Budget Impact, MEA proposal, and a design of the so-called Drug Program (self-contained indication-specific programs). Both pathways undergo rigorous pricing and MEA negotiation process with the Economic Committee, with as many as 4–5 iterations, lasting 4–6 months. The final reimbursement decision is made by the Minister of Health.

The Czech and Polish HTA OMP-specific pathways, as demonstrated throughout this paper, have led to significant improvement in the percentage of EMA-approved medicines being reimbursed. Since this dedicated pathway is specific to the two countries, it is reasonable to assume that it is a critical success factor for efficient and equitable OMP patient access. The only country that bucks this trend is Slovenia, which has the same pathway for all technologies. However, the flexibility in the evidence requirements and decision criteria makes the Slovenian process much closer to Czechia and Poland than any other CEE country, thereby re-confirming that both pre-defined multi-criteria appraisals and flexible criteria processes afford the broadest patient access to OMPs.

## Appendix B. Case Study from Czechia Focusing on Patient Inclusion in Decision-Making Processes

The Czech approach aims at full patient empowerment in pricing and reimbursement decision-making in a two-fold manner: (i) Advisory and data provision to the HTA on all medicines and (ii) voting rights for patient representatives on OMPs in a dedicated MCDA pathway. The first part grants patient representatives true participation, in that patients can submit their preferences and patient-reported outcomes (PROs) in the early stages of the HTA. The second part gives the patients the power to decide, as it equally distributes voting rights (each at 25%) amongst four stakeholders: patients, insurers, clinicians, and the MoH. Additionally, there is also an overarching Patient Council which acts as an advisory body to the Minister of Health on general policies and regulations, rather than specific medicines’ reimbursement. The process by which the Czech system has been reformed took over 4 years and included a step-by-step approach, described in this paper as “5Ds”: (i) Diagnosing/mapping all patient organizations (POs), (ii) defining/proposing optimal PO governance and funding models (independent), (iii) designing/creating a comprehensive educational program for POs, (iv) developing/forming an umbrella PO for rare diseases, and (v) deploying/piloting patient inclusion in decision-making (expert patient representative but not with the disease being appraised). The educational part of the process resulted in the creation of an APO, or Academy of Patients Organization, which continues to be pivotal in equipping patients with the relevant toolset (e.g., terminology and models), robust skillset (e.g., negotiation and debate), and mindset (collaborative multi-stakeholder co-creation). As one of the members of the Patient Council admitted during the interview stage of this paper: “I often speak to fellow patients in different European countries, both in the West and the East. It is clear to me that in Czechia we have by far the most advanced and the most comprehensive system for engaging patients in the HTA and in broader policy-making. It took us a very long time to get to this place, but it was worth it, as our patients are now almost always getting timely access to the golden standard of care”. In this way, the newly created system avoids the key challenges other HTA bodies have identified: tokenism, partiality, and the emotive nature of patient experience data. The Czech model represents a transferable template or a best practice which can be adopted or adapted by virtually all other countries in the region.
